# Creation and validation of a bladder dysfunction symptom score for HTLV-1-associated myelopathy/tropical spastic paraparesis

**DOI:** 10.1186/s13023-020-01451-3

**Published:** 2020-07-03

**Authors:** Natsuko Yamakawa, Naoko Yagishita, Tomohiro Matsuo, Junji Yamauchi, Takahiko Ueno, Eisuke Inoue, Ayako Takata, Misako Nagasaka, Natsumi Araya, Daisuke Hasegawa, Ariella Coler-Reilly, Shuntaro Tsutsumi, Tomoo Sato, Abelardo Araujo, Jorge Casseb, Eduardo Gotuzzo, Steven Jacobson, Fabiola Martin, Marzia Puccioni-Sohler, Graham P. Taylor, Yoshihisa Yamano, the Japan Clinical Research Group on HAM/TSP, Takeo Nakayama, Satoshi Kamei, Jun-ichi Kira, Toshiki Watanabe, Tatsuo Kohriyama, Akihiko Okayama, Atsushi Kawakami, Kenji Yuzawa, Masanori Nakagawa, Tatsufumi Nakamura, Ryuji Kubota, Eiji Matsuura, Koju Kamoi, Takashi Nakajima, Hiroyuki Murai, Kaoru Uchimaru, Yoshio Tsuboi, Yukihiro Namihira, Satoshi Ishihara, Masaaki Niino, Masahiro Nagai, Kunihiko Umekita, Norihiro Takenouchi, Toshio Matsuzaki, Youichi Hokezu, Hideki Nakamura, Takuya Matsushita, Yuji Morio, Hisashi Yonezawa, Takashi Tokashiki, Keiko Tamaki, Hirokuni Sakima, Naoko Yagishita, Tomohiro Matsuo, Junji Yamauchi, Eisuke Inoue, Ayako Takata, Natsumi Araya, Daisuke Hasegawa, Tomoo Sato, Yoshihisa Yamano

**Affiliations:** 1 Department of Rare Diseases Research, Institute of Medical Science, St. Marianna University School of Medicine, Kawasaki, Japan; 2 Department of Neurology, Tokai Central Hospital, Kakamigahara, Japan; 3 Department of Urology, Nagasaki University Graduate School of Biochemical Sciences, Nagasaki, Japan; 4 Department of Medical Informatics, St. Marianna University School of Medicine, Kawasaki, Japan; 5 Department of Preventive Medicine, St. Marianna University School of Medicine, Kawasaki, Japan; 6 Department of Oncology, Karmanos Cancer Institute/Wayne State University, Detroit, MI USA; 7 Department of Advanced Medical Innovation, St. Marianna University Graduate School of Medicine, Kawasaki, Japan; 8 Laboratory for Clinical Research in Neuroinfections, Evandro Chagas National Institute of Infectious Diseases, FIOCRUZ, Rio de Janeiro, Brazil; 9 Institute of Tropical Medicine of Sau Paulo, Sao Paulo, SP Brazil; 10 Instituto de Medicina Tropical “Alexander von Humbldt”, Universidad Peruana Cayetano Heredia, Lima, Peru; 11 Viral immunology Section, Neuroimmunology Branch, National Institute of Neurological Disorders and Stroke, National Institutes of Health, Bethesda, MD USA; 12 Faculty of Medicine, University of Queensland, 288 Herston Road, Herston, QLD 4006 Australia; 13 Escola de Medicina e Cirurgia da Universidade Federal do Estado do Rio de Janeiro/ Universidade Federal do Rio de Janeiro, Rio de Janeiro, Brazil; 14 Section of Virology, Department of Medicine, Imperial College London, St Mary’s Campus, Norfolk Place, London, W2 1PG UK

**Keywords:** Human T-cell leukemia virus type 1, Human T-cell leukemia virus type 1-associated myelopathy/tropical spastic paraparesis, Neurogenic bladder, Urinary symptom score, Bladder dysfunction

## Abstract

**Background:**

Urinary dysfunction is one of the main features of human T-cell leukemia virus type 1-associated myelopathy/tropical spastic paraparesis (HAM/TSP). However, a comprehensive assessment of the severity is difficult because a standardized assessment measure is unavailable. Therefore, this study aimed to develop a novel symptom score for the assessment of urinary dysfunction in HAM/TSP. We interviewed 449 patients with HAM/TSP using four internationally validated questionnaires for assessment of urinary symptoms (27 question items in total): the International Prostate Symptom Score; the International Consultation on Incontinence Questionnaire-Short Form; the Overactive Bladder Symptom Score; and the Nocturia Quality-of-Life questionnaire. We developed a symptom score based on the data of 322 patients who did not use urinary catheters by selecting question items from questionnaires focused on descriptive statistics, correlation analysis, and exploratory factor analysis. The score distribution, reliability, and validity of the developed score were evaluated.

**Results:**

First, 16 questions related to quality of life, situations, or subjective assessment were omitted from the 27 questions. Exploratory factor analysis revealed that the remaining 11 questions pertained to three factors: frequent urination, urinary incontinence, and voiding symptoms. Three questions, which had similar questions with larger factor loading, were deleted. Finally, we selected eight question items for inclusion in the novel score. The score distribution exhibited no ceiling or floor effect. The Cronbach’s alpha (0.737) demonstrated reliable internal consistency. The new score comprised two subscales with acceptable factorial validity (inter-factor correlation coefficient, 0.322): storage symptoms (frequent urination plus urinary incontinence) and voiding symptoms. The correlation between each item and the subscales suggested acceptable construct validity.

**Conclusions:**

We developed a novel score, the HAM/TSP-Bladder Dysfunction Symptom Score, and demonstrated its reliability and validity. The applicability of this score to patients using catheters should be examined in future research.

## Background

Human T-cell leukemia virus type 1 (HTLV-1) is a human retrovirus that has infected at least 5–10 million people worldwide [1, 2]. Approximately 0.3–3% of HTLV-1-infected individuals develop a debilitating disease called HTLV-1-associated myelopathy/tropical spastic paraparesis (HAM/TSP) [3–5]. The primary neuropathological feature of HAM/TSP is chronic meningomyelitis of the white and gray matter, which is followed by axonal degeneration that preferentially affects the lateral funiculus of the spinal cord, particularly at the middle-to-lower thoracic levels [6]. Because the spinal cord is the primary target, the main symptoms of HAM/TSP are spastic paraparesis, neurogenic bladder and bowel dysfunction, and sensory disturbances in the lower limbs.

Up to 90% of patients with HAM/TSP develop neurogenic bladder dysfunction, which is characterized by storage (increased daytime frequency, nocturia, urgency, and urinary incontinence) and/or voiding symptoms (slow stream, intermittent stream, straining, and feeling of incomplete emptying) [7–13]. Patients with HAM/TSP may require intermittent catheterization or indwelling urinary catheters for worsening bladder function, which can severely impact their quality of life (QOL) [12, 14]. The characterization of bladder dysfunction associated with HAM/TSP may vary among individual patients [7–14]. Therefore, clinicians should select suitable medications according to symptoms and determine the potential indication for a urinary catheter, and a comprehensive assessment is necessary to ensure appropriate treatment. However, an accurate assessment of complex urinary symptoms in a clinical setting is challenging because of the lack of standardized assessment measures for patients with HAM/TSP. Moreover, urinary dysfunction has not been assessed comprehensively in this patient population [15, 16]. Therefore, the development of a valid and standardized score for the evaluation of bladder dysfunction severity in HAM/TSP is a key research imperative.

In this study, we aimed to develop a novel symptom score for the assessment of urinary dysfunction in patients with HAM/TSP using data stored in the national HAM/TSP registry (HAM-net) in Japan [17]. This registry includes data pertaining to bladder dysfunction symptoms from approximately 450 patients assessed using the following four internationally validated scores of urinary symptoms: Overactive Bladder Symptom Score (OABSS) [18], International Consultation on Incontinence Questionnaire-Short Form (ICIQ-SF) [19], International Prostate Symptom Score (I-PSS) [20], and Nocturia Quality-of-Life (N-QOL) questionnaire [21]. These scores are used to evaluate frequent urination, urinary incontinence, dysuria, and QOL effects of nocturia, respectively. Here, we developed a novel score, the HAM/TSP-Bladder Dysfunction Symptom Score (HAM-BDSS), by extracting the indispensable items from the above scores and evaluated the validity and reliability of this new score.

## Methods

### Patients

A total of 453 patients registered in the HAM-net between April 1, 2012 and December 31, 2015 were included in this study (UMIN000028400). The HAM-net, which was introduced in March 2012 at St. Marianna University in Japan, is a national registration system for patients with HAM/TSP. Approximately one-fifth or one-sixth of the estimated number of patients in Japan are included in this registry [17]. The HAM-net recruits patients throughout Japan by distributing informational leaflets to patients at clinics and group meetings, as well as to board-certified neurologists in Japan. Patients who wish to register can apply directly to the registration center via telephone, fax, or e-mail. Trained nurses or coordinators conduct annual telephone interviews with the patients in a uniform manner. The data collected from the patients include demographic information, such as sex, age, and financial status, and medical conditions associated and unassociated with HTLV-1 infection. Motor disability is assessed using the Osame Motor Disability Score (OMDS, Table S1), which was established for HAM/TSP [17]. A wide range of neurogenic bladder and lower urinary tract symptoms are evaluated using the Japanese versions of OABSS (4 items, 0–15 points; higher scores indicate more severe status; Table S2) [18]; ICIQ-SF (4 items, 0–21 points; higher scores indicate more severe status; Table S3) [19]; I-PSS (7 items, 0–35 points; higher scores indicate more severe status; Table S4) [20], which is validated for both men and women [22], and N-QOL (12 items, 0–100 points; higher scores indicate better QOL; Table S5) [21]. These urinary scores have been validated for the general population of native Japanese speakers.

This study analyzed the data of 449 patients who responded to the interview. Of the data on the responses to the bladder symptom questionnaires, 1.5% were missing. Patients were classified into four groups based on their dependency on urinary catheters (Table 1): group A, 322 patients who were able to urinate without the use of intermittent or indwelling urinary catheters; group B, 11 patients who were able to urinate but required intermittent catheterization; group C, 104 patients who were not able to urinate and used intermittent catheters; and group D, 12 patients who required the continuous use of indwelling catheters. This study was approved by the St. Marianna University School of Medicine Bioethics Committee (Approval ID No. 2044). All participants in this study provided written informed consent.

**Table 1:** Background characteristics of the study population

	All patients *N* = 449	Group A *N* = 322	Group B *N* = 11	Group C *N* = 104	Group D *N* = 12
sex (Male/Female)	111/338	89/233	0/11	20/84	2/10
Age (mean ± SD)	61.9 ± 10.6	69.0 ± 14.5	58.1 ± 8.3	63.4 ± 9.4	68.3 ± 6.7
age at onset (mean ± SD)	44.7 ± 14.7	44.8 ± 14.4	46.0 ± 10.9	43.4 ± 15.9	52.2 ± 13.1
years from onset (mean ± SD)	17.3 ± 11.3	16.6 ± 11.1	12.1 ± 9.0	20.0 ± 11.9	16.1 ± 8.6
OMDS (mean ± SD) (median [interquartile range])	5.8 ± 2.3 5.0 [5.0–6.0]	5.4 ± 2.1 5.0 [4.0–6.0]	5.9 ± 2.5 5.0 [4.5–6.0]	6.6 ± 2.3 6.0 [5.0–8.0]	9.1 ± 2.9 9.5 [6.0–12.0]
OABSS (mean ± SD) (median [interquartile range])	6.3 ± 4.1 6.0 [3.0–10.0]	6.7 ± 4.0 7.0 [3.0–10.0]	4.6 ± 3.0 5.0 [2.0–7.0]	5.1 ± 4.4 4.0 [2.0–8.0]	
ICIQ-SF (mean ± SD) (median [interquartile range])	6.3 ± 6.0 6.0 [0.0–11.0]	6.5 ± 5.9 7.0 [0.0–11.0]	7.7 ± 5.8 8.0 [2.0–13.5]	5.6 ± 6.4 0.0 [0.0–11.0]	8.7 ± 6.2 12.0 [6.0–13.0]
I-PSS (mean ± SD) (median [interquartile range])	14.2 ± 9.3 14.0 [6.0–22.0]	16.4 ± 8.5 17.0 [10.0–23.0]	12.5 ± 8.2 10.0 [7.0–17.5]	7.4 ± 8.5 3.0 [1.0–12.0]	
N-QOL (mean ± SD) (median [interquartile range])	85.8 ± 17.7 93.8 [77.1–100.0]	85.2 ± 17.9 91.7 [77.1–100.0]	80.7 ± 23.9 93.8 [60.5–100.0]	86.6 ± 16.7 95.8 [74.5–100.0]	100.0 ± 0.0 100.0 [100.0–100.0]

Group A: patients who can urinate by themselves without requiring intermittent catheterization or use of indwelling urinary catheters

Group B: patients who can urinate by themselves but require intermittent catheterization

Group C: patients who cannot urinate by themselves and use intermittent catheters

Group D: patients requiring continued use of indwelling catheters

OMDS: Osame Motor Disability Score

### Evaluation of urinary dysfunction with the four international scores

To investigate whether OABSS, ICIQ-SF, I-PSS, and N-QOL are useful for assessment of the severity of bladder dysfunction in patients with HAM/TSP, the score distributions of the four scores were analyzed.

### Development of HAM-BDSS

To develop a novel score, HAM-BDSS, for assessment of HAM/TSP-related bladder dysfunction symptoms, we extracted question items from the four international scores using the following methodology. To circumvent the effect of catheterization on the source data, the interview survey data of patient group A alone were used.

First, to exclude items subject to the ceiling or floor effect, the distribution of scores measured by each of the four international scores was analyzed. Second, items that fulfilled the following criteria were excluded: those that reflected the situation but did not score the severity of bladder dysfunction (e.g., When does urine leak?), those that depended on a subjective assessment of the respondents (e.g., How much urine do you usually leak?), and those with contents identical to the contents of another item. Next, exploratory factor analysis was performed to determine the common factors among the question items. Furthermore, we calculated Spearman’s rank correlation coefficient to assess the correlation between question items; then, we identified the pairs of items that exhibited correlation coefficient of ≥0.4. Among these pairs, in the order of the highest correlation to the lowest, we selected items by omitting the item with the smaller factor loading in the factor analysis. Once an item was selected or omitted, the decision was considered final.

### Evaluation of the score distribution

To evaluate whether HAM-BDSS appropriately reflects the severity of bladder symptoms in patients with HAM/TSP, we analyzed the distribution of total HAM-BDSS scores in the patient group A. To evaluate the applicability of HAM-BDSS to patients with HAM/TSP who use intermittent or indwelling catheters, we also analyzed the distributions of total HAM-BDSS scores in groups B and C.

### Evaluation of reliability

The internal consistency of HAM-BDSS was assessed by calculating the Cronbach’s α coefficient, which evaluates how closely related a set of items are as a group. The reliability of HAM-BDSS was determined using Cronbach’s α coefficient of ≥0.7. Additionally, Cronbach’s α coefficient was recalculated after omitting each question in HAM-BDSS to examine whether any question had a negative influence on the internal consistency. To circumvent the effect of catheterization, the interview survey data of group A alone were used for the evaluation of reliability.

### Evaluation of validity

We evaluated content validity by investigating the distributions of the selected items according to the classification of the lower urinary tract symptoms defined by the International Continence Society, which is typically used to classify urinary disorder symptoms [23]. For this purpose, we used the definition of each international score described in the original paper to identify the question item that corresponded to each lower urinary tract symptom [18, 20]. We performed exploratory factor analysis to evaluate factorial validity. A factor loading of > 0.30 was considered significant. In addition, Spearman’s rank correlation coefficients were calculated between the question items of HAM-BDSS to evaluate the correlation between each item and the subscales. Correlation coefficients of > 0.7, 0.7–0.4, and < 0.4 were considered to indicate strong, moderate, and weak correlations, respectively. To circumvent the effects of catheterization, the interview survey data of group A alone were used to evaluate the validity.

### Expert opinion

The process of development and the final version of the novel score were discussed and approved by Japanese and international experts on HAM/TSP including neurologists and urologists at the consensus meeting of the Japan Clinical Research Group on HAM/TSP (Oct 2016) and an open workshop held in Kamakura, Japan, during the 18th International Conference on Human Retrovirology: HTLV and related viruses (March 2017).

### Statistical analysis

Medians and interquartile ranges were calculated for describing the four international scores and their distribution was examined graphically. Spearman’s rank correlation coefficient was used to detect a moderate correlation (defined as ≥0.4) between the question items. Exploratory factor analysis was performed using the maximum likelihood method and Promax rotation with Kaiser’s normalization. The number of factors was determined by the Guttman–Kaiser criterion [24, 25]. The factor analysis was also used to evaluate the validity of HAM-BDSS. All data analyses were performed using IBM SPSS Version 22.0 (IBM Corp, Armonk, NY).

## Results

### Evaluation of urinary dysfunction with four international scores

**Fig. 1:**
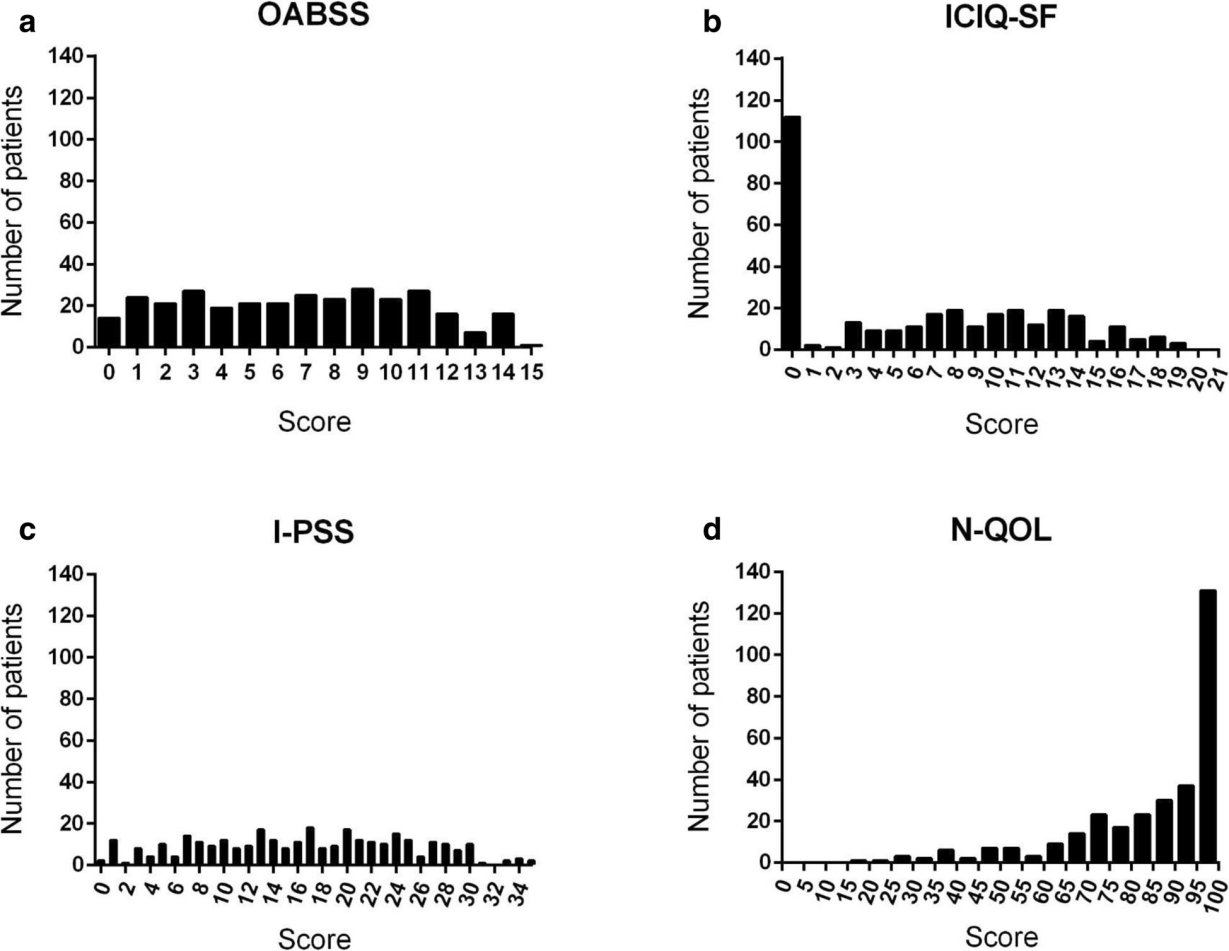
Distribution of the international scores related to urinary symptoms. The distributions of the total scores in group A (patients who do not require intermittent catheterization or use indwelling urinary catheters). **a** Overactive Bladder Symptom Score (OABSS) (*n* = 313); (**b**) International Consultation on Incontinence Questionnaire-Short Form (ICIQ-SF) (*n* = 316); (**c**) International Prostate Symptom Score (I-PSS) (n = 314); and (**d**) the Nocturia Quality-of-Life questionnaire (N-QOL) (*n* = 316)

Patient characteristics and the total scores for the four international scores are summarized in Table 1. Urinary dysfunction is generally considered more severe in order of group A, B, C, and D. However, the total scores for each score did not conform to this order of severity: the total scores tended to be better in groups with more severe urinary dysfunction (Table 1 and Figure S1). We assessed the distributions of the total scores in group A to exclude the influence of catheters (Fig. 1); those of OABSS and I-PSS were uniformly distributed; however, that of ICIQ-SF showed a tendency to converge to 0 and that of N-QOL was skewed toward 100.

### Selection of question items to develop HAM-BDSS

The score distributions for each question item are shown in Figures S2–S5. Most of the scores for questions in OABSS (Figure S2), ICIQ-SF (Figure S3), and I-PSS (Figure S4) were widely distributed; however, the scores for Q3 in ICIQ-SF (Figure S3) and all questions in N-QOL (Figure S5), which are related to patient QOL, were heavily skewed. Therefore, the questions related to QOL were considered not useful for evaluation of the severity of HAM/TSP-related bladder dysfunction symptoms and were excluded from subsequent analyses. This reduced the number of question items from 27 to 14.

Second, we excluded question items that fulfilled the following criteria: items that reflect the situation but do not score the severity of bladder dysfunction (ICIQ-SF Q4, Table S3); items that depend on subjective assessment of the respondents (ICIQ-SF Q2, Table S3). OABSS Q2 (Table S2) was also excluded because it is almost identical to I-PSS Q7 (Table S4) and contains less response items. Consequently, the number of question items was reduced from 14 to 11.

Next, exploratory factor analysis of the 11 items was performed, and divided into three factors (Table 2). Factor 1 had a strong influence on OABSS Q3 and Q4, ICIQ-SF Q1, and I-PSS Q4: items reflecting urinary incontinence. Factor 2 had a strong influence on I-PSS Q2 and Q7 and OABSS Q1: items reflecting frequent urination. Factor 3 had a strong influence on I-PSS Q1, Q3, Q5, and Q6: items reflecting voiding symptoms. The correlation coefficients between factors were low or moderate (0.262, 0.395, 0.432).

**Table 2:** Exploratory factor analysis of international scores

Question item	Factor 1	Factor 2	Factor 3
OABSS Q4	**1.029**	−0.071	− 0.027
ICIQ-SF Q1	**1.022**	−0.065	−0.054
OABSS Q3	**0.658**	0.145	0.083
I-PSS Q4	**0.591**	0.170	0.074
I-PSS Q2	0.000	**0.956**	−0.024
OABSS Q1	0.005	**0.541**	−0.130
I-PSS Q7	0.147	**0.328**	0.050
I-PSS Q5	−0.016	−0.047	**0.778**
I-PSS Q3	−0.033	0.027	**0.706**
I-PSS Q6	0.082	−0.170	**0.494**
I-PSS Q1	−0.004	0.254	**0.342**
Correlation between factors	Factor 1	Factor 2	Factor 3
Factor 1		0.395	0.262
Factor 2	0.395		0.432
Factor 3	0.262	0.432	

The numerical values indicate the strength of the influence of each factor on each question item and the correlation coefficient between factors

We also assessed the correlation between question items by calculating the Spearman’s rank correlation coefficient (Table S6) and identified the pairs of items that showed correlation coefficient of ≥0.4 (Table S7). Among these pairs, OABSS Q1, I-PSS Q4, and ICIQ-SF Q1 were omitted because these items had smaller factor loading in the factor analysis (Table 2).

We finally determined eight question items for the novel score (Table 3). The total score was calculated by adding the score for each question item (0–5), resulting in scores ranging between 0 and 40 points.

**Table 3:** HAM/TSP-bladder dysfunction symptom score (HAM-BDSS)

No	Symptom	Not at All	Less than 1 in 5 times	Less than half the time	About half the time	More than half the time	Almost always
1	In the past month, how often have you had to urinate less than every 2 h?	0	1	2	3	4	5
		None	1 time	2 times	3 times	4 times	5 or more times
2	In the past month, how many times did you typically get up at night to urinate?	0	1	2	3	4	5
		Not at all	Less than once a week	Once a week or more	About once a day	2–4 times a day	5 times a day or more
3	In the past week, how often do you have a sudden desire to urinate, which was difficult to defer?	0	1	2	3	4	5
4	In the past week, how often do you leak urine because you could not defer the sudden desire to urinate?	0	1	2	3	4	5
Score of storage symptoms							/20
		Not at all	Less than 1 in 5 times	Less than half the time	About half the time	More than half the time	Almost always
5	In the past month, how often have you had the sensation of not completely emptying your bladder?	0	1	2	3	4	5
6	In the past month, when urinating, how often have you found yourself to have stopped and then resumed several times?	0	1	2	3	4	5
7	In the past month, how often have you had a weak urinary stream?	0	1	2	3	4	5
8	In the past month, how often have you had to strain to start urination?	0	1	2	3	4	5
Score of voiding symptoms							/20
Total score							/40

Q1 is from I-PSS Q2; Q2 is from I-PSS Q7; Q3 is from OABSS Q3; Q4 is from OABSS Q4; Q5 is from I-PSS Q1; Q6 is from I-PSS Q3; Q7 is from I-PSS Q5; and Q8 is from I-PSS Q6

### Evaluation of the score distribution

**Fig. 2:**
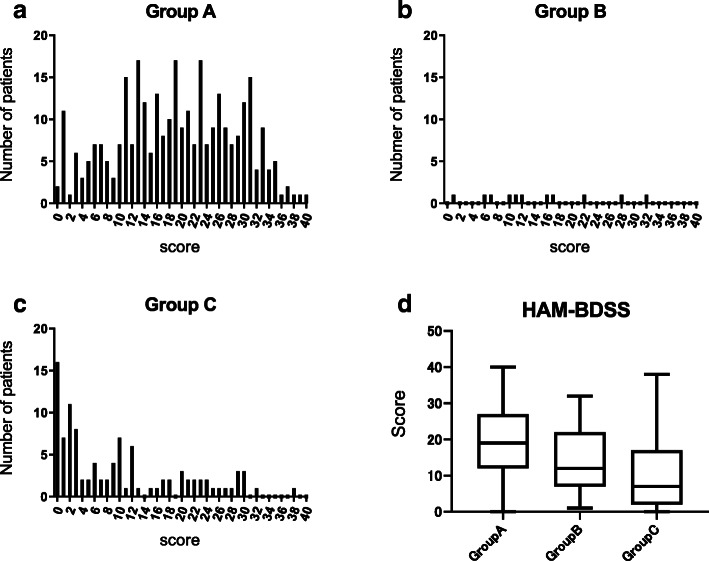
Distribution of the HAM/TSP-bladder dysfunction symptom score (HAM-BDSS) among patients with HAM/TSP. Distribution of HAM-BDSS scores. **a** Group A (patients who do not require intermittent catheterization or use of indwelling urinary catheters, *n* = 314), (**b**) group B (patients who require intermittent catheterization and show control of urine release, n = 11), (**c**) group C (patients who require intermittent catheterization and lacked control of urine release, n = 101). **d** Box plots of HAM-BDSS scores of group **a**, **b**, and **c**

The total scores of HAM-BDSS in group A (*n* = 314) were widely distributed from 0 to 40 points (Fig. 2a). The score distribution in group B was not skewed, although the number of patients was small (*n* = 11, Fig. 2b). However, the score distribution in group C (*n* = 101) was skewed toward lower scores (Fig. 2c). The distribution of scores in group D could not be evaluated. Similarly to the four international scores, the total score of HAM-BDSS showed a tendency to be better in groups with more severe urinary dysfunction (Fig. 2d).

### Evaluation of reliability

Cronbach’s α coefficient of HAM-BDSS was 0.737, which indicated acceptable internal consistency. Additionally, Cronbach’s α coefficient was calculated after omitting each question item in HAM-BDSS (Table S8). If a question item was less relevant than the other question items, the Cronbach’s α for each omitted question item would increase. However, none of the items yielded a value exceeding 0.737 after removal, which suggested the reliability of all question items.

### Evaluation of validity

To determine the content validity, we investigated the distribution of the items in HAM-BDSS according to the classification of the lower urinary tract symptoms defined by the International Continence Society (Table S9) [23]. The question items of HAM-BDSS included most of the defined symptoms, suggesting good content validity. Subsequently, we performed an exploratory factor analysis to evaluate the factorial validity; two factors, Q1–Q4 and Q5–Q8, were extracted (Table 4). The correlation coefficient between the factors was 0.322. Because Q1–Q4 reflect storage symptoms and Q5–Q8 reflect voiding symptoms, this analysis indicated that the factors were appropriately extracted, and that the factorial validity was acceptable. We also analyzed Spearman’s rank correlation coefficient between the question items of HAM-BDSS (Table 5). The correlation strength between each item and the subscale (storage or voiding symptoms) to which the item belongs was moderate or strong; however, the correlation between items and the unrelated subscale was weak, suggesting acceptable construct validity.

**Table 4:** Exploratory factor analysis of HAM/TSP-bladder dysfunction symptom score (HAM-BDSS)

Question item	Factor1	Factor2
Q3	**0.955**	−0.019
Q4	**0.773**	−0.059
Q1	**0.332**	0.304
Q2	**0.323**	0.139
Q6	−0.055	**0.759**
Q7	0.027	**0.748**
Q5	0.177	**0.399**
Q8	0.043	**0.395**
Correlation between factors	0.322	

The numerical values indicate the strength of the influence of each factor on each question item and the correlation coefficient between factors

**Table 5:** Spearman’s rank correlation coefficient between question items in HAM/TSP-bladder dysfunction symptom score (HAM-BDSS)

		Storage symptom				Voiding symptom				Correlation with storage symptoms	Correlation with voiding symptoms
		Q1	Q2	Q3	Q4	Q5	Q6	Q7	Q8		
Storage symptom	Q1		0.392	0.408	0.314	0.359	0.279	0.255	0.071	0.702	0.350
	Q2	0.392		0.347	0.264	0.168	0.148	0.131	0.068	0.624	0.185
	Q3	0.408	0.347		0.731	0.279	0.156	0.200	0.148	0.851	0.269
	Q4	0.314	0.264	0.731		0.165	0.118	0.133	0.133	0.784	0.193
Voiding symptom	Q5	0.359	0.168	0.279	0.165		0.286	0.340	0.184	0.332	0.630
	Q6	0.279	0.148	0.156	0.118	0.286		0.545	0.283	0.236	0.760
	Q7	0.255	0.131	0.200	0.133	0.340	0.545		0.304	0.241	0.760
	Q8	0.071	0.068	0.148	0.133	0.184	0.283	0.304		0.145	0.632

## Discussion

HAM/TSP causes various urinary symptoms, however, there has been no verified score to comprehensively assess these symptoms for this patient population. In this study, we developed a novel score for assessment of HAM/TSP-related bladder dysfunction symptoms by extracting question items from 27 questions of four international scores and demonstrated its reliability and validity. HAM-BDSS is a quantitative score composed of only 8 items, which makes it a more convenient tool for evaluating the severity of bladder symptoms in patients with HAM/TSP.

We used four urinary symptom scores that have been validated in many languages, including Japanese, as a question pool to develop a score that can comprehensively evaluate various urinary symptoms in HAM/TSP. OABSS evaluates the storage symptoms, ICIQ-SF evaluates only urinary incontinence among storage symptoms, and I-PSS evaluates voiding and storage symptoms excluding urinary incontinence. We incorporated 6 of the 7 items from I-PSS in HAM-BDSS. This is because I-PSS is the only score that includes items for voiding as well as storage symptoms. However, I-PSS does not include items that evaluate urinary urgency and incontinence, common symptoms of HAM/TSP-related bladder dysfunction. By combining two questions for these common symptoms included in OABSS, we developed the new score that offers more balanced assessment of bladder dysfunction in HAM/TSP.

In this study, we also demonstrated the reliability and validity of our novel score. The eight questions in HAM-BDSS were well-balanced with respect to the list of urinary symptoms established by the International Continence Society (Table S7) and comprised two subscales to address storage symptoms (Q1–Q4) and voiding symptoms (Q5–Q8). Both types of symptoms are caused by HAM/TSP-related bladder dysfunction and must be evaluated [7–14]. The calculation of the scores for both subscales may help identify the symptoms that cause the greatest degree of impairment. Medications for HAM/TSP-related bladder dysfunction are prescribed depending on the type and severity of symptoms. Therefore, HAM-BDSS scores may also facilitate better treatment decision-making and enable the tailoring of therapy according to the patient’s symptoms.

**Fig. 3:**
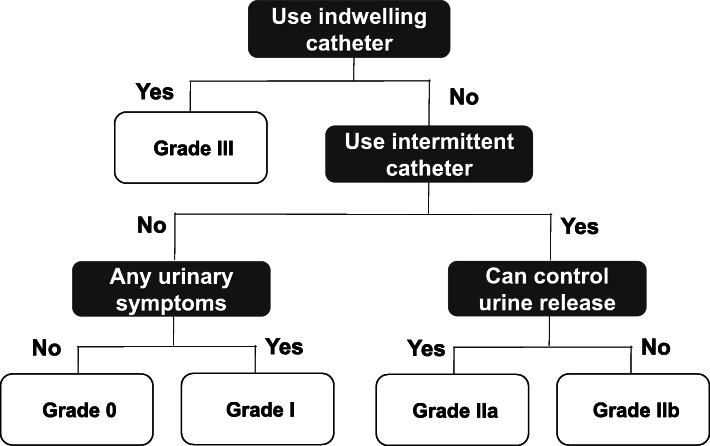
HAM/TSP-bladder dysfunction severity grade (HAM-BDSG). Grading of patients into four severity categories. Patients who use indwelling urinary catheters are classified as grade III; those who require intermittent catheterization and lack urine release control are classified as grade IIb; those who use intermittent catheters and have urine release control are classified as grade IIa; and those who do not use intermittent or indwelling urinary catheters and have or lack urinary symptoms are classified as grade I and 0, respectively

HAM-BDSS scores were widely distributed in patients who can urinate by themselves (groups A and B); however, HAM-BDSS scores of patients who cannot urinate without intermittent catheterization (group C) were skewed toward lower values. Moreover, similarly to the international scores, the scores of HAM-BDSS in patients who need catheters were better than those in patients who can urinate without catheters (Fig. 2). These findings suggest that catheterization improves urinary symptoms but prevents a symptom-based evaluation of bladder dysfunction. Because a substantial proportion of patients with HAM/TSP (approximately 30% of patients in this study) use urinary catheters, both function-based and symptom-based assessments are required for the practical and comprehensive evaluation of HAM/TSP-related bladder dysfunction. Therefore, we would like to propose a grading system, the HAM/TSP-bladder dysfunction severity grade (HAM-BDSG, Fig. 3). HAM-BDSG classifies patients into four grades based on the dependency on urinary catheters, wherein a higher grade indicates greater severity of dysfunction. We expect that the combined use of HAM-BDSG and HAM-BDSS enables objective and comprehensive assessment and treatment decision-making of urinary dysfunction for patients with HAM/TSP.

Neurogenic bladder dysfunction is not only associated with complex symptoms but also with complications such as urinary tract infections and kidney dysfunction. The Neurogenic Bladder Symptom Score (NBSS), which was developed in 2013, was the first score of its kind and has been validated for patients with spinal cord injury, multiple sclerosis, or spina bifida [26, 27]. NBSS comprises 24 questions in three domains (urinary incontinence, bladder storage and voiding, and consequences) as well as additional questions about the bladder dysfunction management and QOL. A short form of NBSS was also reported in 2020 [28]. NBSS includes a question domain regarding consequences (e.g., urinary tract infection, stones, and medication need) in addition to the two question domains included in HAM-BDSS. Future research should validate NBSS for patients with HAM/TSP and compare it with HAM-BDSS. Similar to the urinary symptom scores assessed in the present study, NBSS has been reported to be superior in patients using catheters [29]. This further indicates that the association of a better score with catheter use is a characteristic of the urinary symptom score.

We should acknowledge the limitations of the present study. We developed HAM-BDSS using data of Japanese patients who did not use urinary catheters. Therefore, the applicability of this score for patients using urinary catheters, as well as its international generalizability, need to be examined. Its test–retest reliability should also be evaluated to determine the ability of this score to measure disease progression. For additional verification of the performance of HAM-BDSS, evaluation of its concurrent validity according to the correlation with external criteria, such as frequency volume chart, urodynamic evaluation (including pressure-flow study and post-void residual urine volume) as well as its sensitivity to capture treatment-induced changes in disease severity, would be eagerly awaited.

## Conclusions

In this study, we developed a novel evaluation score for bladder dysfunction in patients with HAM/TSP and demonstrated its validity and reliability. HAM-BDSS represents a comprehensive yet practical scoring algorithm for the assessment of the severity of bladder dysfunction symptoms in these patients.

## Supplementary information

**Additional file 1: Table S1.** Osame Motor Disability Score (OMDS). **Table S2.** Overactive Bladder Symptom Score (OABSS). **Table S3.** International Consultation on Incontinence Questionnaire - Short Form (ICIQ-SF). **Table S4.** International Prostate Symptom Score (I-PSS). **Table S5.** Nocturia-Quality of Life (N-QOL). **Table S6.** Spearman’s rank correlation coefficient between question items in the international scores. **Table S7.** List of question items with a rank correlation coefficient of ≥0.4. **Table S8.** Cronbach’s α-values after the exclusion of each item of the HAM-bladder dysfunction symptom score. **Table S9.** Distribution of the question items of HAM-bladder dysfunction symptom score according to the classification of lower urinary tract symptoms. **Figure S1.** Box plot of total scores of the international urinary scores in various groups. **Figure S2.** Frequency distribution of the responses to each question item of the Overactive Bladder Symptom Score (OABSS) in group A. **Figure S3.** Frequency distribution of responses to each question item of the International Consultation on Incontinence Questionnaire-Short Form (ICIQ-SF) in group A. **Figure S4.** Frequency distribution of responses to each question item of the International Prostate Symptom Score (I-PSS) in group A. **Figure S5.** Frequency distribution of responses to each question item of the Nocturia Quality-of-Life Questionnaire (N-QOL) in group A.

## Data Availability

The datasets analyzed during the current study are available from the corresponding author on reasonable request.

## Abbreviations

HAM-BDSG: HAM/TSP-bladder dysfunction severity grade
HAM-BDSS: HAM/TSP-bladder dysfunction symptom score
HAM/TSP: HTLV-1-associated myelopathy/tropical spastic paraparesis
HTLV-1: Human T-cell leukemia virus type 1
ICIQ-SF: International Consultation on Incontinence Questionnaire-Short Form
I-PSS: International Prostate Symptom Score
NBSS: Neurogenic Bladder Symptom Score
N-QOL: Nocturia Quality-of-Life
OABSS: Overactive Bladder Symptom Score
OMDS: Osame Motor Disability Score
QOL: quality of life
