# Supplementary material for: Creation and validation of a bladder dysfunction symptom score for HTLV-1-associated myelopathy/tropical spastic paraparesis
Source: Orphanet J Rare Dis. 2020 Jul 3;15:175. doi: 10.1186/s13023-020-01451-3 (PMC7333329; doi:10.1186/s13023-020-01451-3)
Supplement: Supplementary file 1 — Additional file 1: Table S1. Osame Motor Disability Score (OMDS). Table S2. Overactive Bladder Symptom Score (OABSS). Table S3. International Consultation on Incontinence Questionnaire - Short Form (ICIQ-SF). Table S4. International Prostate Symptom Score (I-PSS). Table S5. Nocturia-Quality of Life (N-QOL). Table S6. Spearman’s rank correlation coefficient between question items in the international scores. Table S7. List of question items with a rank correlation coefficient of ≥0.4. Table S8. Cronbach’s α-values after the exclusion of each item of the HAM-bladder dysfunction symptom score. Table S9. Distribution of the question items of HAM-bladder dysfunction symptom score according to the classification of lower urinary tract symptoms. Figure S1. Box plot of total scores of the international urinary scores in various groups. Figure S2. Frequency distribution of the responses to each question item of the Overactive Bladder Symptom Score (OABSS) in group A. Figure S3. Frequency distribution of responses to each question item of the International Consultation on Incontinence Questionnaire-Short Form (ICIQ-SF) in group A. Figure S4. Frequency distribution of responses to each question item of the International Prostate Symptom Score (I-PSS) in group A. Figure S5. Frequency distribution of responses to each question item of the Nocturia Quality-of-Life Questionnaire (N-QOL) in group A. [file 13023_2020_1451_MOESM1_ESM.docx]

**Supplementary materials**

**Table S1. Osame Motor Disability Score (OMDS)**

| Grade | Motor disability |
| --- | --- |
| 0 | No walking or running abnormalities |
| 1 | Normal gait but runs slowly |
| 2 | Abnormal gait (stumbling, stiffness) |
| 3 | Unable to run |
| 4 | Needs handrail to climb stairs |
| 5 | Needs a cane (unilateral support) to walk |
| 6 | Needs bilateral support to walk |
| 7 | Can walk 5–10 m with bilateral support |
| 8 | Can walk 1–5 m with bilateral support |
| 9 | Cannot walk, but able to crawl |
| 10 | Cannot crawl, but able to move using arms |
| 11 | Cannot move around, but able to turn over in bed |
| 12 | Cannot turn over in bed |
| 13 | Cannot even move toes |

**Table S2. Overactive Bladder Symptom Score (OABSS)**

| No | Question | Answer | Score |
| --- | --- | --- | --- |
| 1 | How many times do you typically urinate from waking in the morning until sleeping at night? | ≤ 7 | 0 |
|  |  | 8-14 | 1 |
|  |  | ≥ 15 | 2 |
| 2 | How many times do you typically wake up to urinate from sleeping at night until waking in the morning? | 0 | 0 |
|  |  | 1 | 1 |
|  |  | 2 | 2 |
|  |  | ≥ 3 | 3 |
| 3 | How often do you have a sudden desire to urinate, which is difficult to defer? | Not at all | 0 |
|  |  | Less than once a week | 1 |
|  |  | Once a week or more | 2 |
|  |  | About once a day | 3 |
|  |  | 2-4 times a day | 4 |
|  |  | 5 times a day or more | 5 |
| 4 | How often do you leak urine because you cannot defer the sudden desire to urinate? | Not at all | 0 |
|  |  | Less than once a week | 1 |
|  |  | Once a week or more | 2 |
|  |  | About once a day | 3 |
|  |  | 2-4 times a day | 4 |
|  |  | 5 times a day or more | 5 |

In OABSS, patients were instructed to circle the score that best applied to their urinary condition during the past week; the overall score was the sum of the four scores.

**Table S3. International Consultation on Incontinence Questionnaire-Short Form (ICIQ-SF)**

| No | Question | Answer | Score |
| --- | --- | --- | --- |
| 1 | How often do you leak urine?  (Tick one box) | never | 0 |
|  |  | about once a week or less often | 1 |
|  |  | two or three times a week | 2 |
|  |  | about once a day | 3 |
|  |  | several times a day | 4 |
|  |  | all the time | 5 |
| 2 | We would like to know how much urine you think leaks. How much urine do you usually leak (whether you wear protection or not)?　(Tick one box) | none | 0 |
|  |  | a small amount | 2 |
|  |  | a moderate amount | 4 |
|  |  | a large amount | 6 |
| 3 | Overall, how much does leaking urine interfere with your everyday life?  Please ring a number between 0 (not at all) and 10 (a great deal) |  | 0 |
|  |  |  | 1 |
|  |  |  | 2 |
|  |  |  | 3 |
|  |  |  | 4 |
|  |  |  | 5 |
|  |  |  | 6 |
|  |  |  | 7 |
|  |  |  | 8 |
|  |  |  | 9 |
|  |  |  | 10 |
| 4 | When does urine leak?  (Please tick all that apply to you) | never - urine dose not leak |  |
|  |  | leaks before you can get to the toilet |  |
|  |  | leaks when you cough or sneeze |  |
|  |  | leaks when you are asleep |  |
|  |  | leaks when you are physically active/exercising |  |
|  |  | leaks when you have finished urinating and are dressed |  |
|  |  | leaks for no obvious reason |  |
|  |  | leaks all the time |  |

**Table S4. International Prostate Symptom Score (I-PSS)**

| No | Question | Answer | Score |
| --- | --- | --- | --- |
| 1 | In the past month, how often have you had the sensation of not emptying your bladder? | Not at All | 0 |
|  |  | Less than 1 in 5 Times | 1 |
|  |  | Less than Half the Time | 2 |
|  |  | About Half the Time | 3 |
|  |  | More than Half the Time | 4 |
|  |  | Almost Always | 5 |
| 2 | In the past month, how often have you had to urinate less than every two hours? | Not at All | 0 |
|  |  | Less than 1 in 5 Times | 1 |
|  |  | Less than Half the Time | 2 |
|  |  | About Half the Time | 3 |
|  |  | More than Half the Time | 4 |
|  |  | Almost Always | 5 |
| 3 | In the past month, how often have you found you stopped and started again several times when you urinated? | Not at All | 0 |
|  |  | Less than 1 in 5 Times | 1 |
|  |  | Less than Half the Time | 2 |
|  |  | About Half the Time | 3 |
|  |  | More than Half the Time | 4 |
|  |  | Almost Always | 5 |
| 4 | In the past month, how often have you found it difficult to postpone urination? | Not at All | 0 |
|  |  | Less than 1 in 5 Times | 1 |
|  |  | Less than Half the Time | 2 |
|  |  | About Half the Time | 3 |
|  |  | More than Half the Time | 4 |
|  |  | Almost Always | 5 |
| 5 | In the past month, how often have you had a weak urinary stream? | Not at All | 0 |
|  |  | Less than 1 in 5 Times | 1 |
|  |  | Less than Half the Time | 2 |
|  |  | About Half the Time | 3 |
|  |  | More than Half the Time | 4 |
|  |  | Almost Always | 5 |
| 6 | In the past month, how often have you had to strain to start urination? | Not at All | 0 |
|  |  | Less than 1 in 5 Times | 1 |
|  |  | Less than Half the Time | 2 |
|  |  | About Half the Time | 3 |
|  |  | More than Half the Time | 4 |
|  |  | Almost Always | 5 |
| 7 | In the past month, how many times did you typically get up at night to urinate? | Not at All | 0 |
|  |  | Less than 1 in 5 Times | 1 |
|  |  | Less than Half the Time | 2 |
|  |  | About Half the Time | 3 |
|  |  | More than Half the Time | 4 |
|  |  | Almost Always | 5 |

**Table S5. Nocturia Quality of Life (N-QOL)**

| No | Question | Answer | Score |
| --- | --- | --- | --- |
| 1 | Over the past 2 weeks, having to get up at night to urinate has made it difficult for me to concentrate the next day | Every day | 0 |
|  |  | Most days | 1 |
|  |  | Some days | 2 |
|  |  | Rarely | 3 |
|  |  | Never | 4 |
| 2 | Over the past 2 weeks, having to get up at night to urinate has made me feel generally low in energy the next day | Every day | 0 |
|  |  | Most days | 1 |
|  |  | Some days | 2 |
|  |  | Rarely | 3 |
|  |  | Never | 4 |
| 3 | Over the past 2 weeks, having to get up at night to urinate has required me to nap during the day | Every day | 0 |
|  |  | Most days | 1 |
|  |  | Some days | 2 |
|  |  | Rarely | 3 |
|  |  | Never | 4 |
| 4 | Over the past 2 weeks, having to get up at night to urinate has made me less productive the next day | Every day | 0 |
|  |  | Most days | 1 |
|  |  | Some days | 2 |
|  |  | Rarely | 3 |
|  |  | Never | 4 |
| 5 | Over the past 2 weeks, having to get up at night to urinate has caused me to participate less in activities I enjoy | Extremely | 0 |
|  |  | Quite a bit | 1 |
|  |  | Moderately | 2 |
|  |  | A little bit | 3 |
|  |  | Not at all | 4 |
| 6 | Over the past 2 weeks, having to get up at night to urinate has caused me to be careful about when or how much I drink | All the time | 0 |
|  |  | Most of the time | 1 |
|  |  | Some of the time | 2 |
|  |  | Rarely | 3 |
|  |  | Never | 4 |
| 7 | Over the past 2 weeks, having to get up at night to urinate has made it difficult for me to get enough sleep at night | Every night | 0 |
|  |  | Most nights | 1 |
|  |  | Some nights | 2 |
|  |  | Rarely | 3 |
|  |  | Never | 4 |
| 8 | Over the past 2 weeks, I have been concerned that I am disturbing others in the house because of having to get up at night to urinate | Extremely | 0 |
|  |  | Quite a bit | 1 |
|  |  | Moderately | 2 |
|  |  | A little bit | 3 |
|  |  | Not at all | 4 |
| 9 | Over the past 2 weeks, I have been preoccupied about having to get up at night to urinate | All the time | 0 |
|  |  | Most of the time | 1 |
|  |  | Some of the time | 2 |
|  |  | Rarely | 3 |
|  |  | Never | 4 |
| 10 | Over the past 2 weeks, I have been worried that this condition will get worse in the future | Extremely | 0 |
|  |  | Quite a bit | 1 |
|  |  | Moderately | 2 |
|  |  | A little bit | 3 |
|  |  | Not at all | 4 |
| 11 | Over the past 2 weeks, I have been worried that there is no effective treatment for this condition (having to get up at night to urinate) | Extremely | 0 |
|  |  | Quite a bit | 1 |
|  |  | Moderately | 2 |
|  |  | A little bit | 3 |
|  |  | Not at all | 4 |
| 12 | Overall, how bothersome has having to get up at night to urinate been during the past 2 weeks? | Extremely | 0 |
|  |  | Quite a bit | 1 |
|  |  | Moderately | 2 |
|  |  | A little bit | 3 |
|  |  | Not at all | 4 |

In N-QOL, items of Q1 to Q12 were scored on a scale of 0–4 with a greater score indicating better QOL. Scores were then summed and transformed into a standardized scale ranging from 0–100.

**Table S6. Spearman’s rank correlation coefficient between question items in the international scores**

|  | OABSS Q1 | OABSS Q3 | OABSS Q4 | ICIQ-SF Q1 | I-PSS Q1 | I-PSS Q2 | I-PSS Q3 | I-PSS Q4 | I-PSS Q5 | I-PSS Q6 | I-PSS Q7 |
| --- | --- | --- | --- | --- | --- | --- | --- | --- | --- | --- | --- |
| OABSS Q1 |  | 0.194 | 0.133 | 0.137 | 0.067 | 0.484 | 0.111 | 0.169 | 0.076 | 0.059 | 0.110 |
| OABSS Q3 | 0.194 |  | 0.729 | 0.721 | 0.281 | 0.415 | 0.155 | 0.763 | 0.206 | 0.154 | 0.347 |
| OABSS Q4 | 0.133 | 0.729 |  | 0.990 | 0.168 | 0.316 | 0.122 | 0.696 | 0.137 | 0.137 | 0.264 |
| ICIQ-SF Q1 | 0.137 | 0.721 | 0.990 |  | 0.157 | 0.313 | 0.108 | 0.692 | 0.129 | 0.128 | 0.246 |
| I-PSS Q1 | 0.067 | 0.281 | 0.168 | 0.157 |  | 0.360 | 0.289 | 0.267 | 0.343 | 0.187 | 0.168 |
| I-PSS Q2 | 0.484 | 0.415 | 0.316 | 0.313 | 0.360 |  | 0.277 | 0.443 | 0.261 | 0.079 | 0.392 |
| I-PSS Q3 | 0.111 | 0.155 | 0.122 | 0.108 | 0.289 | 0.277 |  | 0.198 | 0.545 | 0.284 | 0.148 |
| I-PSS Q4 | 0.169 | 0.763 | 0.696 | 0.692 | 0.267 | 0.443 | 0.198 |  | 0.228 | 0.207 | 0.338 |
| I-PSS Q5 | 0.076 | 0.206 | 0.137 | 0.129 | 0.343 | 0.261 | 0.545 | 0.228 |  | 0.308 | 0.131 |
| I-PSS Q6 | 0.059 | 0.154 | 0.137 | 0.128 | 0.187 | 0.079 | 0.284 | 0.207 | 0.308 |  | 0.068 |
| I-PSS Q7 | 0.110 | 0.347 | 0.264 | 0.246 | 0.168 | 0.392 | 0.148 | 0.338 | 0.131 | 0.068 |  |

**Table S7. List of question items with a rank correlation coefficient of ≥0.4**

| Pair of question items | Rank correlation coefficient | Omitted items | Selected items |
| --- | --- | --- | --- |
| OABSS Q4 | 0.990 | ICIQ-SF Q1 | OABSS Q4 |
| ICIQ-SF Q1 |  |  |  |
| OABSS Q3 | 0.763 | I-PSS Q4 | OABSS Q3 |
| I-PSS Q4 |  |  |  |
| OABSS Q3 | 0.729 | None | OABSS Q3 and OABSS Q4 are  already selected |
| OABSS Q4 |  |  |  |
| OABSS Q3 | 0.721 | ICIQ-SF Q1 is  already omitted | OABSS Q3 is  already selected |
| ICIQ-SF Q1 |  |  |  |
| OABSS Q4 | 0.696 | I-PSS Q4 is  already omitted | OABSS Q4 is  already selected |
| I-PSS Q4 |  |  |  |
| ICIQ-SF Q1 | 0.692 | ICIQ-SF Q1 and I-PSS Q4 are  already omitted | None |
| I-PSS Q4 |  |  |  |
| OABSS Q1 | 0.484 | OABSS Q1 | I-PSS Q2 |
| I-PSS Q2 |  |  |  |
| OABSS Q3 | 0.415 | None | OABSS Q3 and I-PSS Q2 are already selected |
| I-PSS Q2 |  |  |  |

**Table S8. Cronbach's α-values after the exclusion of each item of the HAM-bladder dysfunction symptom score**

| Question item omitted | Cronbach's α without each item |
| --- | --- |
| Q1 | 0.695 |
| Q2 | 0.720 |
| Q3 | 0.688 |
| Q4 | 0.710 |
| Q5 | 0.704 |
| Q6 | 0.694 |
| Q7 | 0.692 |
| Q8 | 0.733 |

**Table S9. Distribution of the question items of HAM-bladder dysfunction symptom score according to the classification of lower urinary tract symptoms**

| Lower urinary tract symptoms defined in ICS | | Question items of the novel index |
| --- | --- | --- |
| Storage symptoms | Increased daytime frequency | Q1 (I-PSS Q2) |
|  | Nocturia | Q2 (I-PSS Q7) |
|  | Urgency | Q3 (OABSS Q3) |
|  | Urinary incontinence | Q4 (OABSS Q4) |
|  | Bladder sensation |  |
| Voiding symptoms | Slow stream | Q7 (I-PSS Q5) |
|  | Splitting or spraying |  |
|  | Intermittent stream | Q6 (I-PSS Q3) |
|  | Hesitancy |  |
|  | Straining | Q8 (I-PSS Q6) |
|  | Terminal dribble |  |
| Post micturition symptoms | Feeling of incomplete emptying | Q5 (I-PSS Q1) |
|  | Post micturition dribble |  |

**Figure S1. Box plot of total scores of the international urinary scores in various groups**

Box plots of scores of (A) Overactive Bladder Symptom Score (OABSS, n = 313 in group A; n= 11 in group B; n = 101 in group C), (B) International Consultation on Incontinence Questionnaire-Short Form (ICIQ-SF, n = 316 in group A; n= 11 in group B; n = 101 in group C; n = 3 in group D), (C) International Prostate Symptom Score (I-PSS, n = 314 in group A; n= 11 in group B; n = 104 in group C), and (D) Nocturia Quality-of-Life questionnaire (N-QOL, n = 316 in group A; n= 11 in group B; n = 101 in group C; n = 3 in group D). Group A comprised patients who were able to urinate themselves without requiring intermittent catheterization or use of indwelling urinary catheters; group B comprised patients who were able to urinate themselves but required intermittent catheterization; group C comprised patients who were unable to urinate by themselves and require intermittent catheterization; group D comprised patients who required continued use of indwelling catheters.


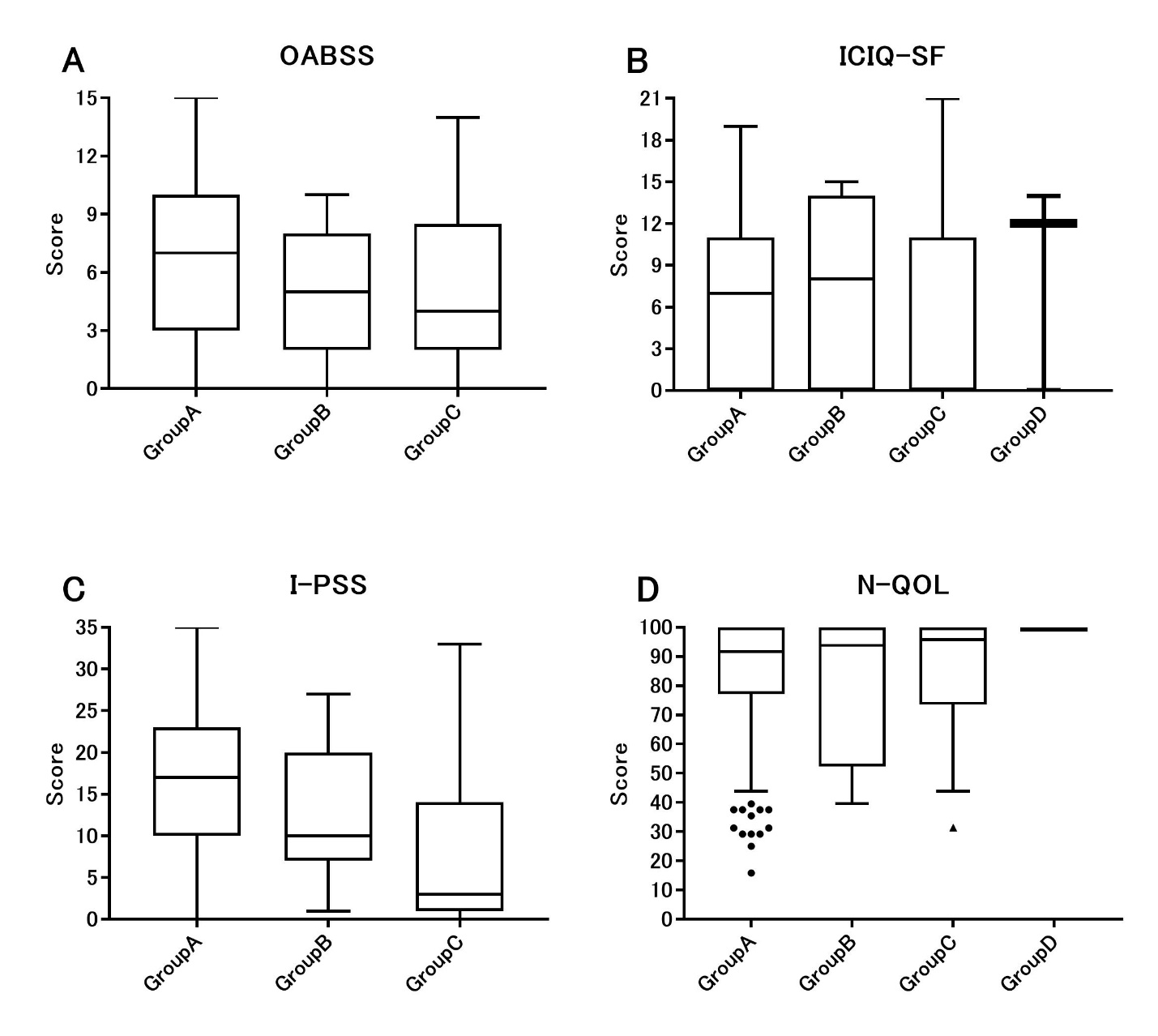


**Figure S2.** **Frequency distribution of the responses to each question item of the Overactive Bladder Symptom Score (OABSS) in group A**

Group A comprised of patients who are able to urinate by themselves without requiring intermittent catheterization or use of indwelling urinary catheters (n = 313). The vertical axes indicate the responses to each question item of OABSS.

**
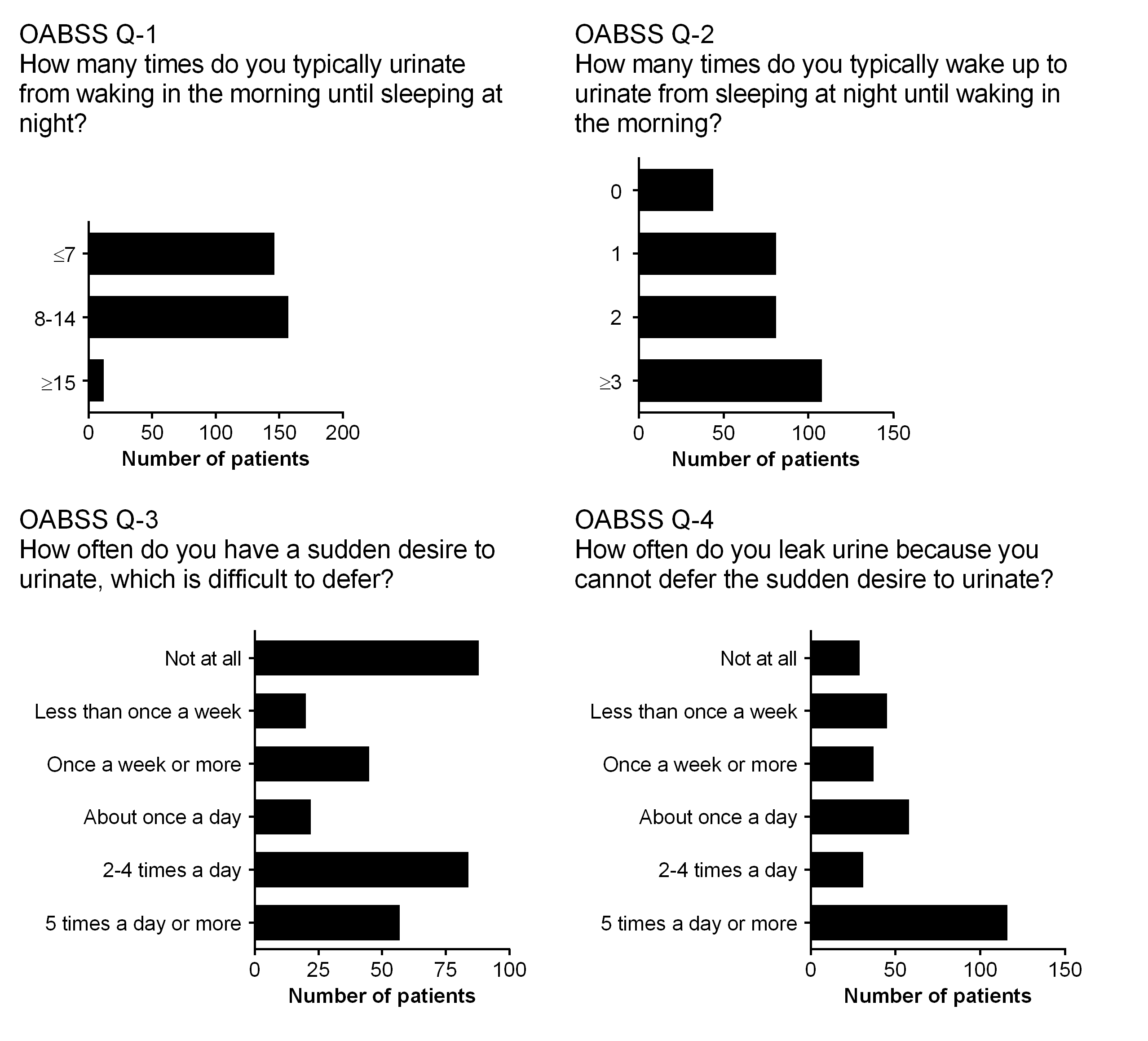
**

**Figure S3. Frequency distribution of responses to each question item of the International Consultation on Incontinence Questionnaire - Short Form (ICIQ-SF) in group A**

Group A comprised of patients who are able to urinate by themselves without requiring intermittent catheterization or use of indwelling urinary catheters (n = 316). The vertical axes indicate responses to each question item of ICIQ-SF.


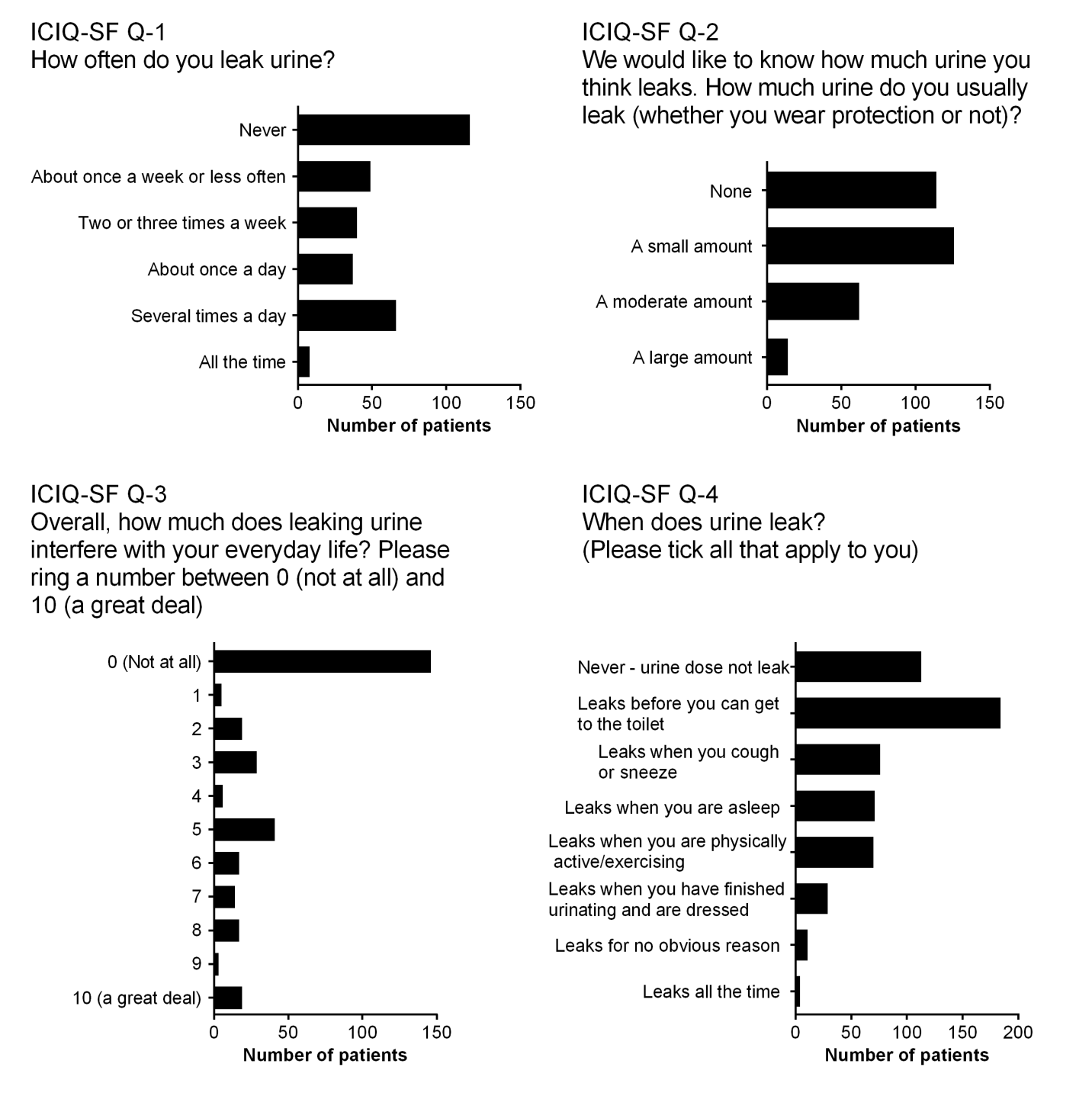


.

**Figure S4. Frequency distribution of responses to each question item of the International Prostate Symptom Score (I-PSS) in group A**

Group A comprised of patients who are able to urinate by themselves without requiring intermittent catheterization or use of indwelling urinary catheters (n = 314). The vertical axes indicate the responses to each question item of I-PSS.


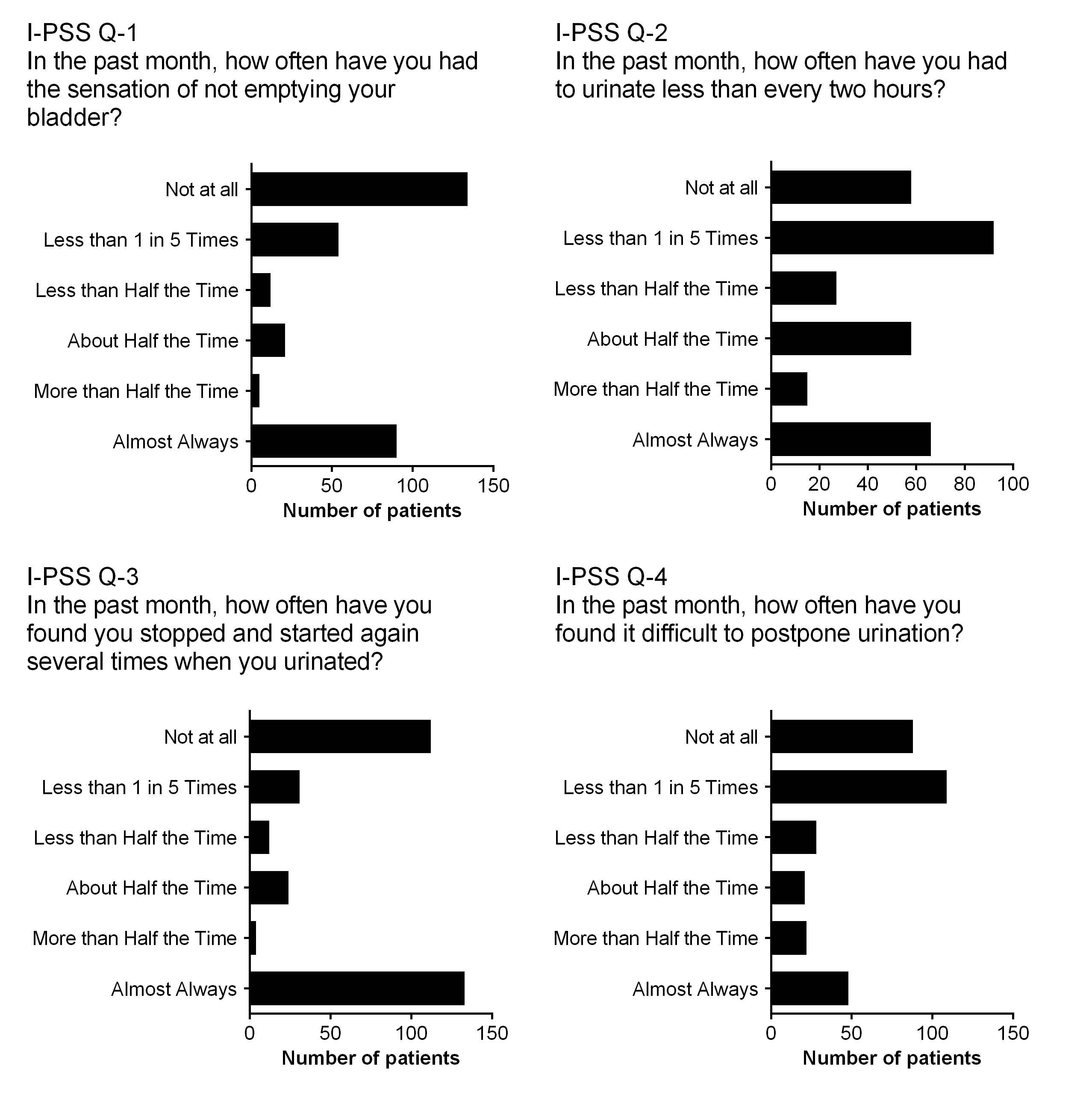


**
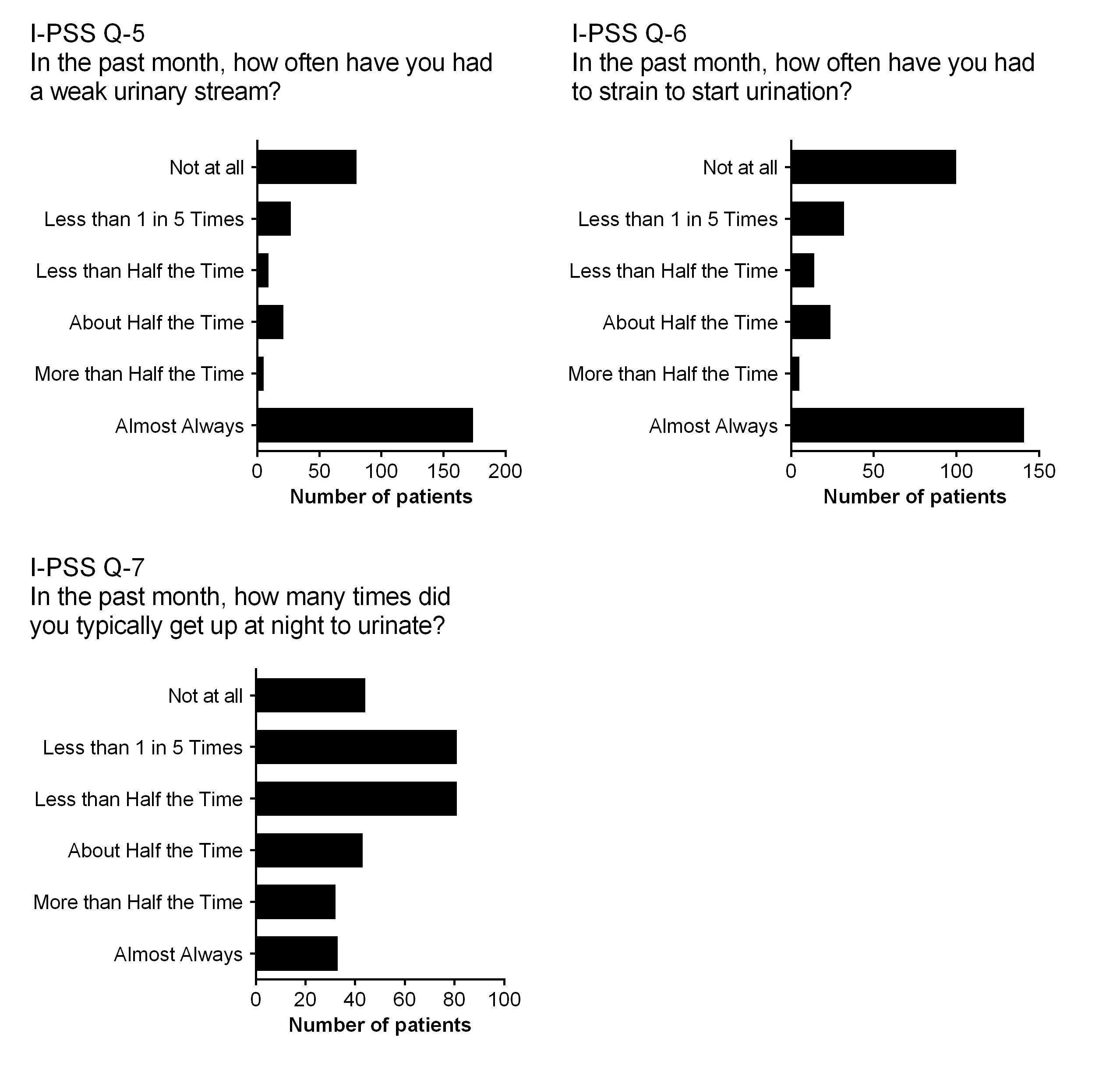
**

**Figure S5. Frequency distribution of responses to each question item of the Nocturia Quality-of-Life Questionnaire (N-QOL) in group A**

Group A comprised of patients who are able to urinate by themselves without requiring intermittent catheterization or use of indwelling urinary catheters (n = 316). The vertical axes indicate the responses to each question item of N-QOL.


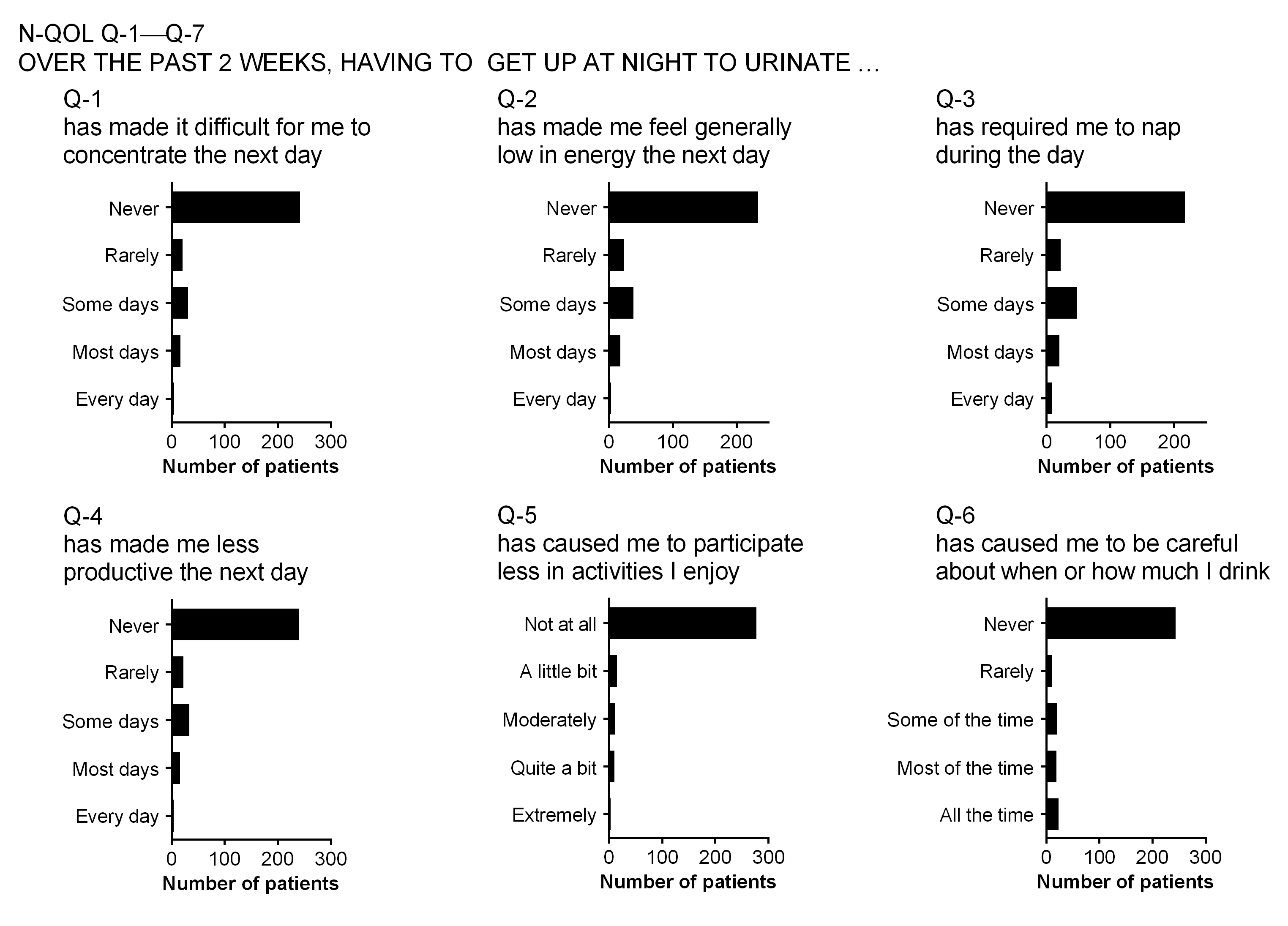

**
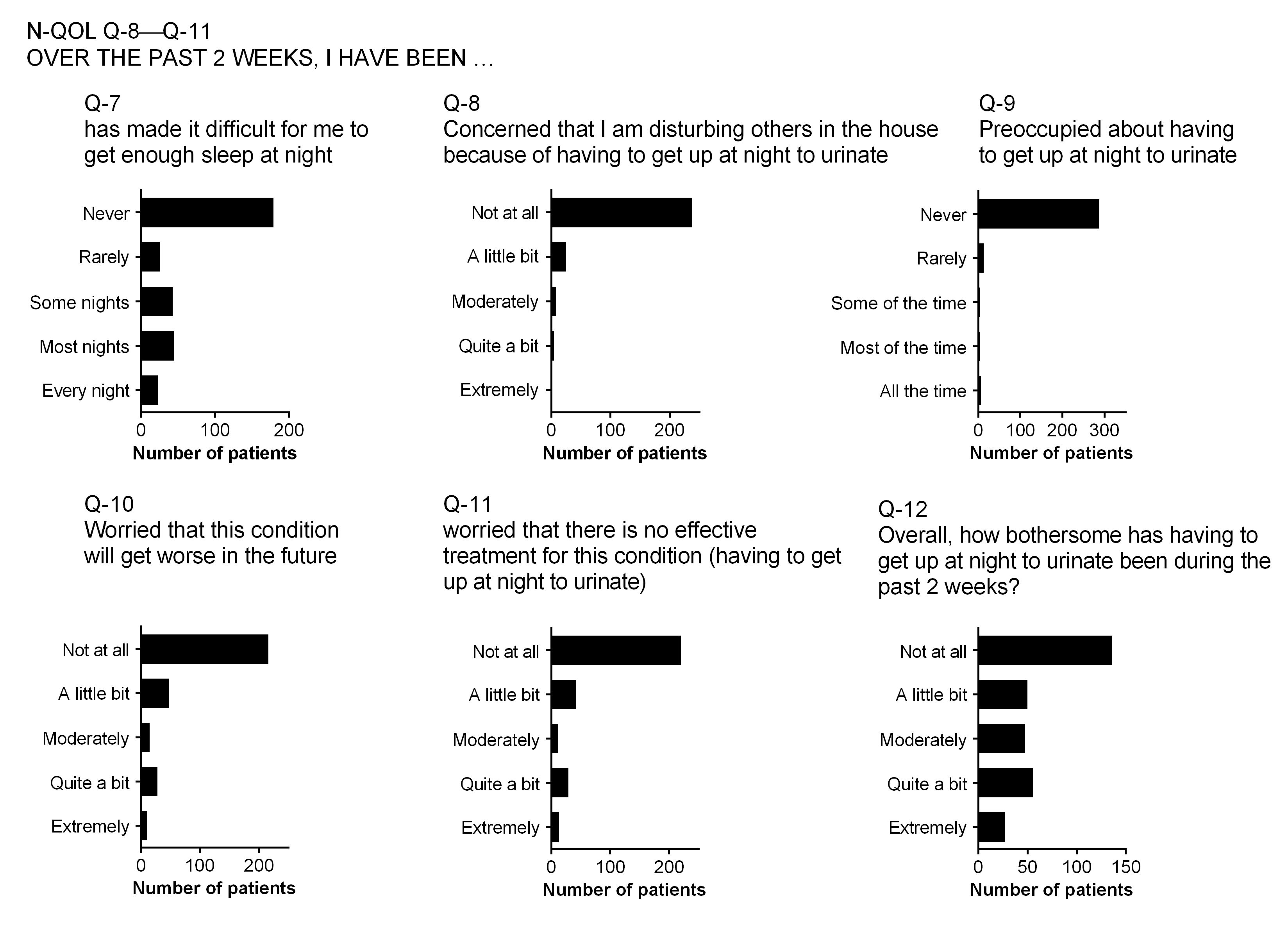
**
